# miRNAs Plasma Profiles in Vascular Dementia: Biomolecular Data and Biomedical Implications

**DOI:** 10.3389/fncel.2016.00051

**Published:** 2016-03-01

**Authors:** Marco Ragusa, Paolo Bosco, Lucia Tamburello, Cristina Barbagallo, Angelo G. Condorelli, Mariangela Tornitore, Rosario S. Spada, Davide Barbagallo, Marina Scalia, Maurizio Elia, Cinzia Di Pietro, Michele Purrello

**Affiliations:** ^1^Section of Biology and Genetics G Sichel, BioMolecular, Genome and Complex Systems BioMedicine Unit (BMGS), Department of BioMedical Sciences and BioTechnology, University of Catania, Catania, Italy; ^2^Istituto di Ricovero e Cura a Carattere Scientifico (IRCCS) per il Ritardo Mentale e l’Involuzione Cerebrale Senile Oasi Maria SS, Troina, Enna, Italy

**Keywords:** vascular dementia, Alzheimer’s dementia, non-coding-RNAs plasma profiles, liquid biopsies, biomarkers

## Abstract

Vascular dementia (VaD) is a pathogenetically heterogeneous neuropsychiatric syndrome, mainly characterized by cognitive impairment. Among dementias, it is second by incidence after Alzheimer’s dementia (AD). VaD biomolecular bases have been poorly characterized, but vascular-linked factors affecting the CNS and its functions are generally hypothesized to perform a major role, together with cardiovascular and immunological factors. miRNAs, which perform critically important biomolecular roles within cell networks, are also found in biological fluids as circulating miRNAs (cmiRNAs). We hypothesized that differentially expressed (DE) cmiRNAs in plasma from VaD patients could be applied to diagnose VaD through *liquid* biopsies; these profiles also could allow to start investigating VaD molecular bases. By exploiting TaqMan Low-Density Arrays and single TaqMan assays, miR-10b*, miR29a-3p, and miR-130b-3p were discovered and validated as significantly downregulated DE cmiRNAs in VaD patients compared to unaffected controls (NCs). These miRNAs also were found to be significantly downregulated in a matched cohort of AD patients, but miR-130b-3p levels were lower in AD than in VaD. A negative correlation was detected between miR-29a and miR-130b expression and cognitive impairment in VaD and AD, respectively. Receiver operating characteristic curves demonstrated that decreased plasma levels of miR-10b*, miR29a-3p, and miR-130b-3p allow to discriminate VaD and AD patients from NCs. Furthermore, the concurrent downregulation of both miR-10b* and miR-130b-3p in VaD showed an area under the curve (AUC) of 0.789 (*p* < 0.0001) with 75% of sensitivity and 72% of specificity, whereas an AUC of 0.789 (*p* < 0.0001) with 92% of sensitivity and 81% of specificity was found for both in AD. The miRNAs profiles reported in this paper pave the way to translational applications to molecular VaD diagnosis, but they also should allow to further investigate on its molecular bases.

## Introduction

Neurodegenerative diseases (NDs) are a large group of clinically severe and difficult to treat pathological phenotypes, which are characterized by neuronal death in different areas of the brain (Ghavami et al., 2014). Due to an aging world population, it has been forecast that their prevalence will dramatically increase unless strong measures are applied to prevent their *epidemic* diffusion (Hebert et al., 2013; Raz et al., 2015). Dementias are characterized by progressive cognitive decline; they are caused by various pathological processes, including neurodegeneration (Raz et al., 2015). The National Plan to address Alzheimer’s disease classified Alzheimer’s disease-related dementias as (1) Alzheimer’s dementia (AD), (2) vascular dementia (VaD), (3) dementia with Lewy bodies, (4) frontotemporal dementia, and (5) mixed dementias (Jagtap et al., 2015). VaD is further classified as (a) large-vessel VaD, which includes cortical or subcortical multi-infarct dementia and strategic infarct dementia; (b) small vessel VaD, including subcortical ischemic dementia and other forms of dementia due to specific arteriopathies; (c) hemorrhagic dementia; and (d) hypoperfusion VaD (Raz et al., 2015). It has been estimated that between 1 and 4% of people aged 65 years or more are affected by VaD, whose prevalence is predicted to double every 5–10 years past the age of 65 (Raz et al., 2015). Currently, VaD molecular bases have been poorly characterized (Montine et al., 2014). It then ensues that differential diagnosis with other types of dementia as AD is very difficult to perform (Gratten et al., 2014). This represents an important hurdle for developing presymptomatic screening tests and eventually designing personalized therapies. It is common knowledge that most of our genome is composed of genes encoding RNA molecules other than mRNAs: these RNAs, which do not code for proteins, are denominated non-coding RNAs (ncRNAs) and constitute the genome’s *dark matter* (Tay et al., 2014). ncRNAs are classified as long non-coding RNAs (lncRNAs), if their length is >200 nt, or small non-coding RNAs (sncRNAs), if their length is ≤200 nt (Tay et al., 2014). It has been clearly demonstrated that microRNAs (miRNAs, sncRNAs of 22–28 nt) are master regulators of networks and pathways in critically important cellular processes (Tay et al., 2014); accordingly, miRNAs have been shown to be causally involved in neoplastic and degenerative diseases (Geaghan and Cairns, 2014). It also has been discovered that miRNAs are present in blood as circulating miRNAs (cmiRNAs), either as cell-free complexes with RNA-binding proteins (e.g., Ago-2) or enclosed within membrane-bound vesicles (e.g., exosomes) (Jung and Suh, 2014). Since cmiRNAs are traceable in biological fluids as serum, plasma, and cerebrospinal fluid (CSF), it is not surprising that they have been already exploited as molecular biomarkers for diseases affecting CNS (Geekiyanage et al., 2012). To date, this approach has not been applied to VaD: due to the potential importance of this type of data, we sought to characterize plasma miRNAs profiles of VaD patients and to compare them with those from a cohort of patients affected by AD and from matched control individuals (NCs). This allowed the identification of three cmiRNAs (miR-10b*, miR29a-3p, and miR-130b-3p) that are significantly downregulated in VaD, the characterization of their downstream networks, and the identification of a set of target genes that are involved in neurodegeneration and cardiovascular pathology.

## Materials and Methods

### Patient Selection

Vascular dementia and AD patients and age-, sex-, and ethnicity-matched control individuals were recruited at *Istituto Oasi Maria SS. Troina* (Enna, Italy) between January 2000 and December 2010 (Table 1); plasma miRNAs profiles were analyzed at the University of Catania between 2014 and 2015. In total, 118 individuals were selected: 38 VaD, 40 AD, and 40 NCs (Table 1). VaD patients were identified through NINDS-AIREN criteria (McVeigh and Passmore, 2006) and AD patients by NINCDS-ADRDA criteria (McKhann et al., 1984) (Tables S1 and S2 in Supplementary Material). Control individuals (NCs) were hospitalized volunteers who did not present VaD, AD nor were affected by other neurodegenerative, cardiovascular, and neoplastic diseases. Both patients and NCs were Sicilian individuals of Caucasian ethnicity and had a low-average educational attainment. Following formal written consent, patients underwent venipuncture using dry vacutainer tubes; blood samples were centrifuged at 4000 rpm for 15 min at 20°C to isolate plasma, which was subdivided into aliquots and stored at −80°C until analysis. Ethical approval for this study was provided by the Ethics Committee of IRCCS *Associazione Oasi Maria SS*.

**Table 1:** **Demographics of VaD, AD patients, and NCs**.

Samples and controls	Gender		Age		MMSE	
	
	Male	Female	Mean	SD	Mean	SD
VaD	18	20	82.24	6.58	14.14	5.75
AD	17	23	81.375	4.68	19	4.93
NCs	19	21	81.9	6.18	28.5	1.95

### Extraction of miRNAs from Plasma

RNA from 400 μl plasma samples was extracted by using a Qiagen miRNeasy Mini Kit according to Qiagen Supplementary Protocol (Qiagen, GmbH, Hilden, Germany), which allows the purification of small RNAs (including miRNAs) from plasma or serum; RNA was eluted with 30 μl volume of elution buffer and quantified by spectrophotometry (Rizzo et al., 2015).

### RNA Reverse Transcription and Preamplification: miRNAs Profiling by TaqMan Low-Density Array

To profile plasma cmiRNAs in VaD patients, we selected and matched four of them for sex and age with four NCs. Male/female ratio was 1; mean age for VaD was 86.25 and for NCs 87.5; mean Mini-Mental State Examination (MMSE) was 19 for VaD and 25 for NCs. Plasma RNAs (3 μl) were retrotranscribed and preamplified with TaqMan MicroRNA Reverse Transcription Kit Applied Biosystems (primers for the RT Megaplex™ RT Primers, Human Pool A and Pool B), PreAmp Master Mix, and Megaplex PreAmp Primers. We profiled the transcriptome of 754 miRNAs with TaqMan Low-Density Arrays (TLDAs), TaqMan Human MicroRNA Array v3.0 A and B (Applied Biosystems Life Technologies™, Monza, Italy), by utilizing 18 μl of preamplified products. PCRs on TLDAs were performed on a 7900HT Fast Real Time PCR System (Applied Biosystem, Life Technologies™, Monza, Italy).

### TLDAs Data Analysis

To obtain an accurate miRNAs normalization, we used the global median normalization (GMN) method. Similar to microarray analysis, *C*_t_ values from each sample were normalized to the median *C*_t_ of the array (Ragusa et al., 2010). By computing Pearson correlation among *C*_t_ medians and means of each array and *C*_t_ of each miRNA, we identified a miRNA that showed an expression profile close to the median and mean of TLDAs: miR-191-5p. We applied the statistical test significance analysis of microarrays (SAM), included in Mev (Multi experiment viewer v4.8.1) statistical analysis software,^1^ applying a two-class paired and unpaired test among Δ*C*_t_s. A false discovery rate (FDR) <0.15 was chosen as filter. Relative quantity (RQ) of miRNAs was calculated by applying the $2^{-\Delta \Delta C_{\text{t}}}$ method.

### Single TaqMan Assays

To validate data from profiling, specific single assays were applied to differentially expressed (DE) miRNAs among VaD, AD, and NCs, exploiting reverse transcription (Reverse Transcription Kit, Applied Biosystem) and Real Time PCR with TaqMan probes. miR-191-5p was used for normalization by applying the $2^{-\Delta \Delta C_{\text{t}}}$ method.

### Statistical Analysis

All statistical analyses were performed using the MedCalc software (Version 15.11.4). *T*-tests (paired and unpaired) were used to compare miRNAs plasma levels among VaD, AD patients, and NCs. Δ*C*_t_s for DE miRNAs respect to endogenous control miR-191-5p were used to generate a receiver operating characteristic (ROC) curve. Area under the curve (AUC) and 95% confidence intervals (95% CIs) were calculated to assess the accuracy of each parameter (sensitivity and specificity) and to find an appropriate cut-off point. Statistical significance was established at a *p*-value ≤0.05.

### Target Prediction

Validated targets of DE miRNAs were retrieved from miRTarbase, release 4.5.^2^ Target prediction was obtained by intersecting the predictions by Starbase v2.0^3^ and DIANA-microT CDS v5.0.^4^ Among targets predicted by both tools, we selected those showing a miRSVR score ≤−0.1.

### Pathway Enrichment Analysis

Pathway enrichment analysis of validated and predicted targets of DE miRNAs was performed with two different tools: Gene Trail^5^ and DIANA mirPath v2.0.^6^ The *p* values for the biological categories, obtained with the gene set analysis tool GeneTrail, were adjusted by FDR and were considered significant if *p* < 0.05. The functional annotation tool DIANA mirPath v2.0 retrieves both experimentally verified miRNAs targets from DIANA-TarBase v7.0^7^ as predicted miRNAs targets from DIANA-microT-CDS (see text footnote 3); for pathway enrichment analysis with DIANA mirPath, we used only predicted targets by DIANA-microT-CDS as no validated targets were found for miR-10b-3p in DIANA-TarBase. MicroT threshold of 0.8 and *p*-value <0.05 (Benjamini–Hochberg correction) were selected.

### Network Analysis

Selected targets and their nearest neighbors were used to construct an interaction network with MiMi Plugin 3.1^8^ in Cytoscape v2.8.3.^9^ Centrality analysis was performed by CentiScaPe Plugin v.1.21,^10^ where parameters of betweenness, closeness, degree, and stress were selected to identify the most central nodes. To further analyze the biological relevance of nodes, Cytoscape plug-in ClueGO v2.1.5 was used to perform functional enrichment analysis in Gene Ontology and KEGG pathways.

### Target mRNAs Quantification from Plasma

We extracted mRNA from 400 μl plasma by using Trizol purification protocol; mRNAs encoding CCT5, GSK3 (targets of miR-10b*), BACE1, LPL, NAV3 (targets of miR-29a), EDN1, ITPR1, and ZEB-1 (targets of miR-130b) were amplified through Power Sybr Green One-Step Real Time PCR (Life Technologies), following the manufacturer’s protocol. GPDH was used as housekeeping gene. Primers sequences are reported in Table S3 in Supplementary Material.

## Results

### Identification of DE cmiRNAs in VaD

**Figure 1:**
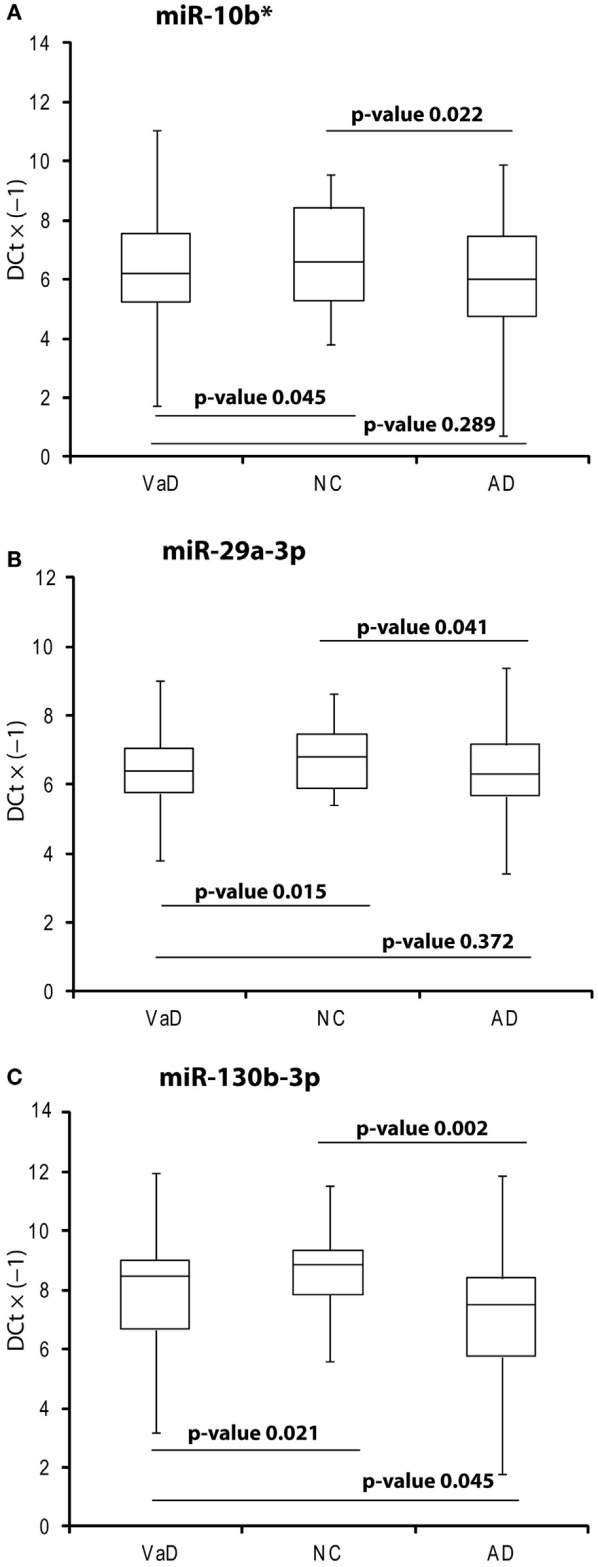
Box plot of miR-10b* (A), miR-29a-3p (B), miR-130b-3p (C) expression. Validation was by single TaqMan assays in VaD patients compared to AD and NCs, and in AD compared to NCs. Values on the *y*-axis are reported as Δ*C*_t_ × (−1). Samples analyzed: 38 VaD, 40 AD, and 40 CN. Statistical significance was evaluated by paired *T*-test.

In the discovery phase of our project, we applied TaqMan Low-Density Array technology to profile the levels of 754 miRNAs in plasma from 4 VaD patients and 4 matched NCs. This led to the identification of 13 potentially significant DE miRNAs (Table 2): among these, we focused our validation analysis on the most dysregulated miRNAs that were endowed with qualitatively good amplification curves. In particular, miR-886-5p and 886-3p showed apparently significant overexpression in VaD compared to NCs, whereas miR-10b* (alternative nomenclature: miR-10b-3p), miR-29a-3p (alternative nomenclature: miR-29a), and miR-130b-3p (alternative nomenclature: miR-130b) showed significant underexpression. In the validation phase of our work, we extended our analysis to the whole cohort of 38 VaD patients and 40 NCs. Single assays for each miRNA confirmed that indeed miR-10b*, miR-29a-3p, and miR-130b-3p are all significantly underexpressed in plasma from VaD patients with respect to NCs (Figure 1). Overexpression of miR-886-5p and miR-886-3p did not stand this further statistical test. Expression of miR-10b*, miR-29a-3p, and miR-130b-3p was then evaluated in plasma from 40 AD patients, matched by gender, age, and ethnic background with the 38 VaD patients and 38 NCs previously analyzed. This allowed us to discover that all three miRNAs were DE in a statistically significant manner among the different cohorts (Figure 1). miR-10b* was underexpressed in VaD and AD compared to NCs, while its plasma levels were not significantly different in the comparison between VaD and AD patients (Figure 1A). A similar expression trend was observed in the analysis of miR-29a-3p for all types of comparison (Figure 1B). On the other hand, miR-130b-3p was significantly underexpressed in VaD and AD plasma compared to NCs, but in AD patients its expression levels were lower than in VaD (Figure 1C). We calculated the mathematical correlation (i.e., Pearson and Spearman coefficients) between miRNAs expression (Δ*C*_t_) and MMSE from patients and healthy controls. Through this analysis, we found a statistically significant negative correlation between miR-29a expression and MMSE in VaD patients (Pearson = −0.28, *p*-value = 0.011; Spearman = −0.23 *p*-value = 0.04), and between miR-130b expression and MMSE in AD patients (Pearson = −0.28, *p*-value = 0.011; Spearman = −0.29 *p*-value = 0.009).

**Table 2:** **List of differential expressed miRNAs in VaD vs. NCs identified by TaqMan Low-Density Arrays**.

miRNAs	RQ
hsa-miR-103	0.18
hsa-miR-10b*	**0.35**
**hsa-miR-130b**	**0.27**
hsa-miR-142-5p	0.57
hsa-miR-143	0.29
hsa-miR-145	0.77
hsa-miR-181c	0.45
hsa-miR-185	2.61
hsa-miR-223	0.48
hsa-miR-26b	0.37
**hsa-miR-29a**	**0.22**
**hsa-miR-886-3p**	**3.47**
**hsa-miR-886-5p**	**3.04**

*In bold are miRNAs selected for Single TaqMan assays. RQ, relative quantity. False discovery rate <0.15*.

### ROC Curves

**Figure 2:**
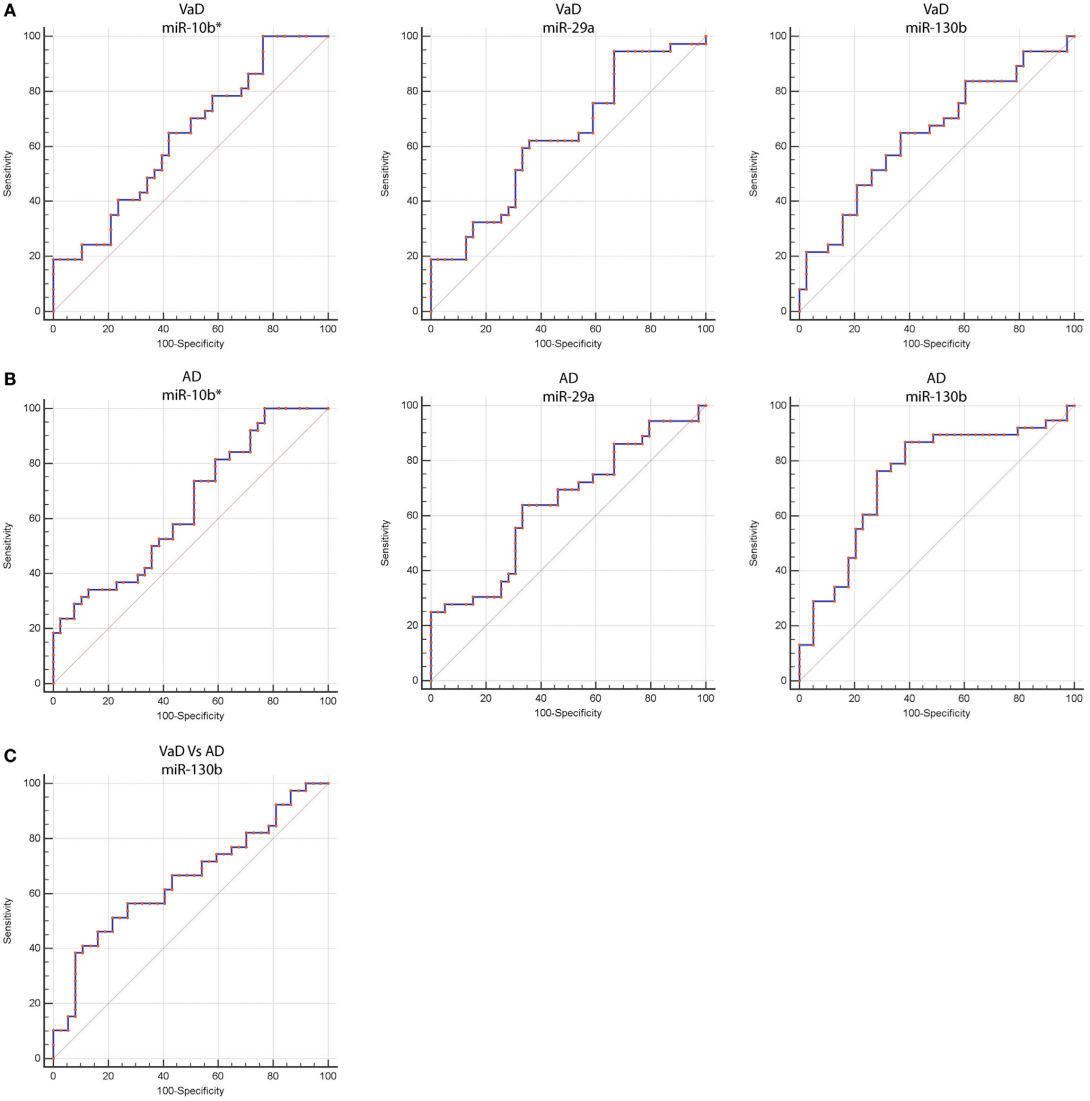
Receiver operating characteristic (ROC) curves for miR-10b*, miR-29a-3p, and miR-130b-3p in VaD and AD patients. ROC curves of miR-10b*, miR-29a-3p, and miR-130b-3p Δ*C*_t_s in VaD **(A)** and AD **(B)**. **(C)** ROC curve of miR-130b-3p Δ*C*_t_s for discrimination of VaD patients from AD ones. Red curve represents Δ*C*_t_s calculated by using miR-191-5p as endogenous control.

**Figure 3:**
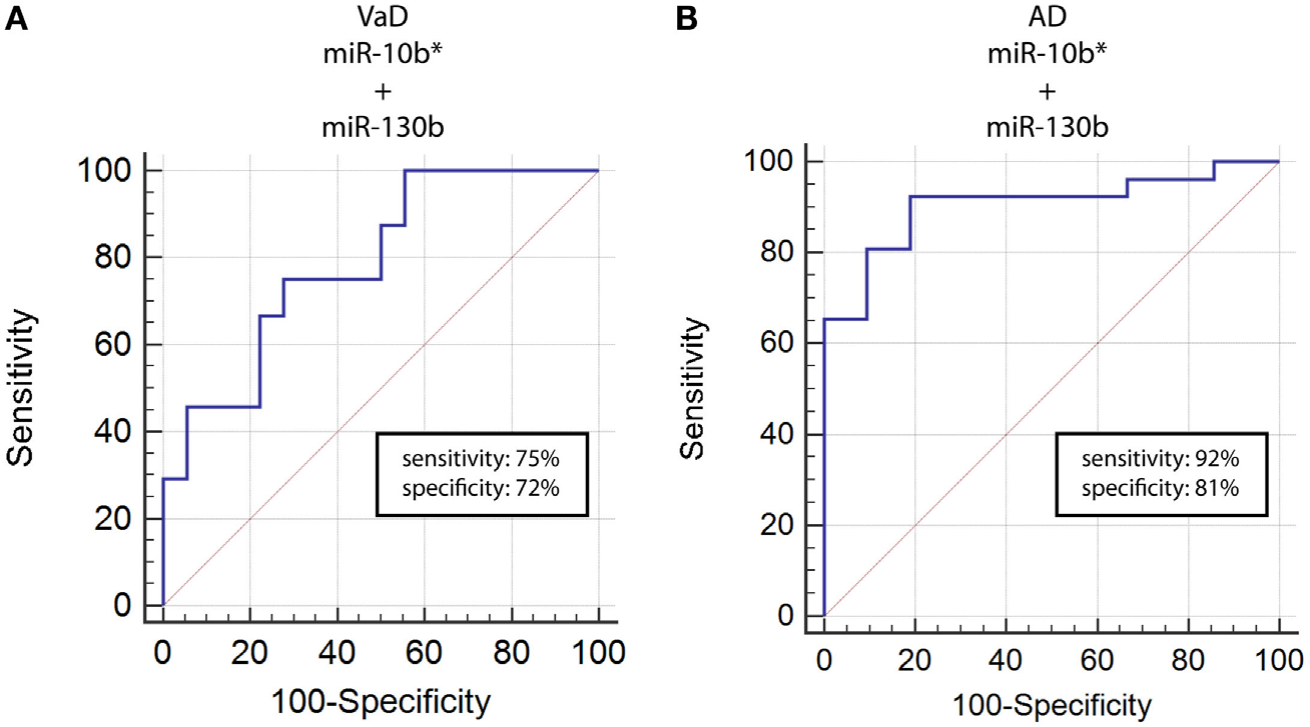
ROC curves for couple miR-10b*–miR-130b-3p in VaD and AD patients. ROC curves computed by considering together miR-10b* and miR-130b-3p Δ*C*_t_s for detecting VaD patients **(A)** and AD patients **(B)**.

By computing ROC curves, we found that the decrease of miR-10b*, miR-29a, and miR-130b plasma levels was able to discriminate VaD and AD patients from NCs. Specifically, we obtained for miR-10b* in VaD, an AUC of 0.63 (95% CI, 0.507–0.759; *p* = 0.03) with 78% of sensitivity and 42% of specificity (Δ*C*_t_ cut-off value: >1.73); for miR-29a in VaD, an AUC of 0.63 (95% CI, 0.507–0.759; *p* = 0.03) with 76% of sensitivity and 41% of specificity (Δ*C*_t_ cut-off value: >1.05); for miR-130b in VaD, an AUC of 0.65 (95% CI, 0.520–0.769; *p* = 0.02) with 68% of sensitivity and 53% of specificity (Δ*C*_t_ cut-off value: >−0.62) (Figure 2A). By performing the same analysis with data from AD patients, we obtained for miR-10b*, an AUC of 0.64 (95% CI, 0.520–0.769; *p* = 0.02) with 74% of sensitivity and 48% of specificity (Δ*C*_t_ cut-off value: >2.14); for miR-29a, an AUC of 0.64 (95% CI, 0.520–0.769; *p* = 0.02) with 75% of sensitivity and 41% of specificity (Δ*C*_t_ cut-off value: >1.05); for miR-130b, an AUC of 0.74 (95% CI, 0.620–0.850; *p* > 0.0001) with 87% of sensitivity and 61% of specificity (Δ*C*_t_ cut-off value: >−0.43) (Figure 2B). Moreover, we calculated a ROC curve also for evaluating the discriminatory power of miR-130b in VaD patients with respect to those with AD: an AUC of 0.65 (95% CI, 0.530–0.780; *p* = 0.01) with 70% of sensitivity and 46% of specificity (Δ*C*_t_ cut-off value: ≤0.91) was obtained (Figure 2C). To improve the potential diagnostic power of DE cmiRNAs, we also computed ROC curves for every pair of cmiRNAs: the best score in terms of sensitivity and specificity was obtained for the couple miR-10b*–miR-130b, which showed for VaD an AUC of 0.789 (95% CI, 0.636–0.90; *p* < 0.0001) with 75% of sensitivity and 72% of specificity (Figure 3A), whereas an AUC of 0.789 (95% CI, 0.783–0.971; *p* < 0.0001) with 92% of sensitivity and 81% of specificity was found for AD (Figure 3B).

### Pathway Analysis

**Figure 4:**
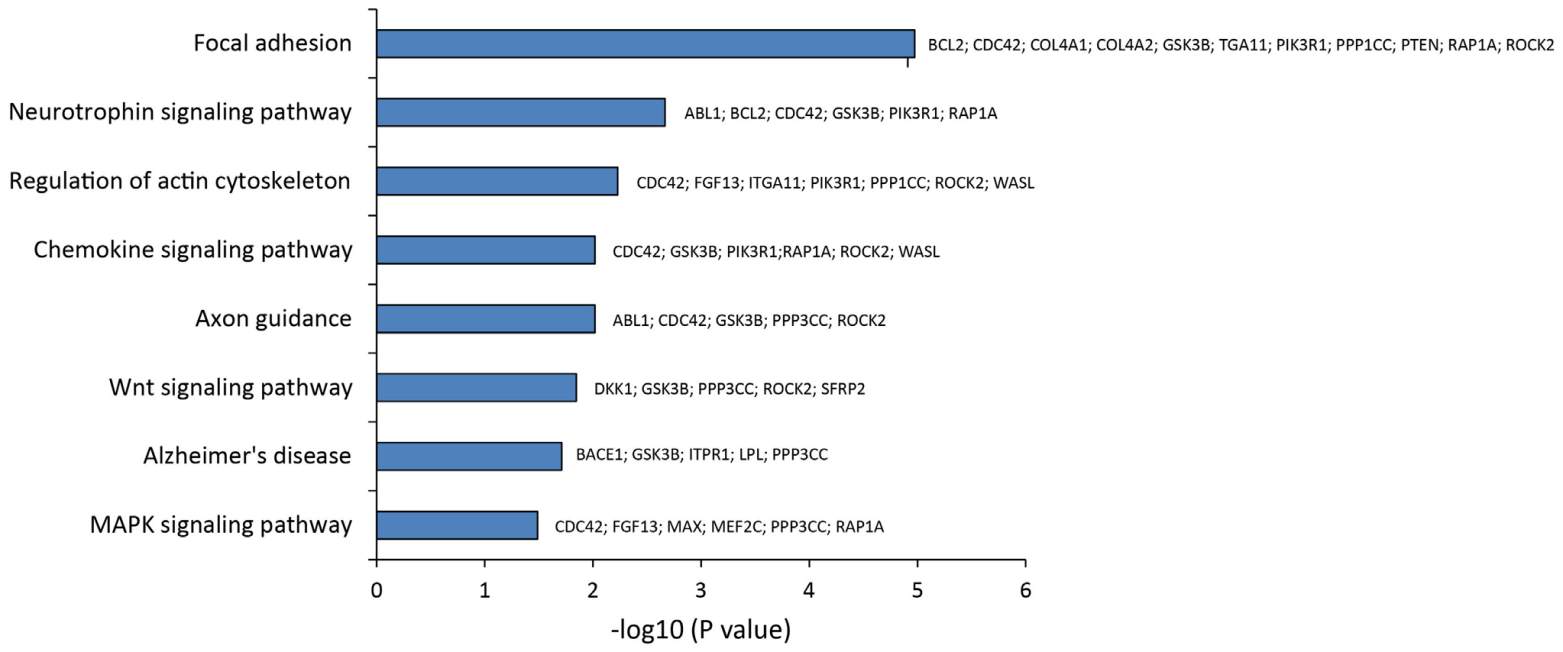
**Statistical overrepresented KEGG pathways, identified by GeneTrail, and target genes included in each KEGG category**.

To pinpoint the biomolecular functions of these DE miRNAs, we analyzed their mRNAs targets. This identified (A) 55 predicted targets of miR-10b*, (B) 43 validated and 9 predicted targets of miR-29a-3p, and (C) 7 validated targets and 14 predicted targets of miR-130b-3p (Table S4 in Supplementary Material). By exploiting CentiScaPe Plugin, we reconstructed a network of 2149 nodes and 23704 edges: among the 225 nodes, which were discovered to be central for all selected parameters, 14 were either validated or putative targets of DE miRNAs (Table S5 in Supplementary Material). Through GeneTrail Pathway Enrichment Analysis, we discovered that target genes of miR-10b*, miR-29a-3p, and miR-130b-3p are enriched in each subcategory of eight non-neoplastic KEGG pathways (Figure 4). Interestingly, the following subcategories resulted overrepresented also by using DIANA mirPath v2.0 functional annotation tool: *axon guidance*, *focal adhesion*, *neurotrophin signaling pathway*, *Wnt signaling pathway* (Figure 2). According to ClueGO, the most involved KEGG pathways are *cell cycle*, *focal adhesion*, *FoxO signaling*, *hepatitis B*, *insulin signaling*, *MAPK signaling*, *neurotrophin signaling*, *Rap1 signaling*, *T cell receptor signaling*; three of these (*focal adhesion*, *MAPK signaling*, and *neurotrophin signaling*) were also identified through GeneTrail. Among the mRNAs targets of miR-10b*, miR-29a-3p, and miR-130b-3p, which were analyzed in plasma from VaD and AD patients and NCs (i.e., BACE1, CCT5, EDN1, GSK3B, ITPR1, LPL, NAV3, and ZEB1), only ZEB1 (target of miR-130b-3p) was detected; however, its plasma levels were not different among the individuals of the three cohorts analyzed.

## Discussion

### miRNAs as VaD Molecular Biomarkers

The important biomolecular roles played by miRNAs within organisms at all levels of the evolutionary scale have been demonstrated (Zheng et al., 2016). Many miRNAs are enriched in specific organs, tissues, and cell types, for instance, in different areas of the brain or in specific subcompartments of neurons (e.g., axons, dendrites, and synapses) (He et al., 2012). cmiRNAs have been detected in plasma, serum, whole blood, urine, saliva, sweat, breath, and cerebrospinal fluid (Li and Zhang, 2015). Because of their small molecular size compared to proteins, cmiRNAs cross biological barriers (e.g., blood/brain, blood/placenta); they also are present within exosomes isolated from body fluids (Li and Zhang, 2015). cmiRNAs have been already exploited as biomarkers for depression, bipolar disorder, schizophrenia, and Alzheimer’s disease (Ha, 2011), but not for VaD. VaD is thought to be caused by diminished cerebral blood flow leading to hypoxia and blood–brain barrier altered permeability: vasculotoxic and neurotoxic effects ensue, which may promote neurodegeneration. VaD may be caused by different pathological events as stroke, cerebral hemorrhage, traumas, chronic diseases as atherosclerosis, large and small vessel disease, and cardioembolic disease (Iadecola, 2013). It is frequently associated with diabetes, hypertension, hypercholesterolemia, and smoking. In contrast to AD, VaD genetic bases are not well defined (Montine et al., 2014). Due to the high compensatory potential of the brain, both AD and VaD are characterized by late clinical manifestations (Raz et al., 2015). This delay clearly calls for early activation of diagnostic presymptomatic and preventive procedures: when the disease becomes clinically evident, pharmacological intervention may no longer be very effective (Hebert et al., 2013; Raz et al., 2015). Diagnostic criteria for AD and VaD, based on clinical evaluation of cognitive decline and deterioration of functional abilities, have been proposed by (1) the Diagnostic and Statistical Manual of Mental Disorders, Fifth Edition (DSM-5) (Regier et al., 2009), (2) the International Classification of Diseases (ICD-10), (3) the USA National Institute of Neurological Disorders and Stroke and the Association Internationale pour la Recherche et l’Enseignement en Neurosciences (NINDS-AIREN) (McVeigh and Passmore, 2006), (4) the MMSE (Folstein et al., 1975), and (5) the California Alzheimer’s Disease Diagnostic and Treatment Centres (CAD-DTC) (Chui et al., 1992). Moreover, the most recent applications of magnetic resonance imaging (MRI) and computed tomography (CT) make possible to analyze in detail the brain structure and confirm the diagnosis of cerebrovascular diseases as VaD (Van Straaten and Stam, 2013). We propose that the molecular data reported in this paper nicely complement the diagnostic approaches synthesized above: in fact, our data show that miR-10b*, miR-29a-3p, and miR-130b-3p are DE in plasma from VaD patients with respect to NCs. They also are underexpressed in plasma from AD patients with respect to NCs, but the levels of miR-130b-3p are lower in AD than in VaD patients (Figure 1B). A negative correlation exists between miR-29a Δ*C*_t_ and MMSE in VaD, as between miR-130b Δ*C*_t_ and MMSE in AD. These data showed that plasma levels of these two miRNAs decreased as the cognitive impairment increased, suggesting a hypothetical link. ROC curve analysis suggests that these miRNAs could be considered useful markers to diagnose VaD and AD. miR-130b levels were also able to discriminate VaD from AD with 70% of sensitivity and 46% of specificity. Intriguingly, by considering the diagnostic efficiency of different pairs of cmiRNAs, we found that the concurrent downregulation of both miR-10b* and miR-130b-3p improved their discriminatory power of VaD and AD patients. Accordingly, miR-10b*, miR-29a-3p, and miR-130b-3p are all to be considered good markers of VaD and AD (and of neurodegeneration in general), but miR-130b-3p may be specifically exploited as a differential diagnostic marker between VaD and AD. We found in literature no previous mention of circulating or cellular miR-10b* related to pathological or physiological conditions. On the other hand, decreased levels of miR-29a in serum and whole blood of AD patients were previously reported (Geekiyanage et al., 2012; Leidinger et al., 2013). Recently, a meta-analysis of cmiRNAs deregulated in AD and MCI from 18 independent published reports was published: the downregulation of miR-29a in serum or plasma from AD patients was reported by four independent papers, and in two of them its deregulation resulted statistically significant (Wu et al., 2015). The authors concluded that the inconsistence of results across different studies should be the consequence of multiple biological and technical factors. miRNAs expression could be influenced by age, genetic variations related to ethnicity, environmental factors, comorbid conditions, and by biological source of miRNAs (e.g., serum or plasma), sample preparation, analysis platform, normalization method, and statistical approaches. Notably, about 40% of papers reporting data on cmiRNAs in AD and MCI were studies on Chinese population (Wu et al., 2015). An altered expression of miR-130b was reported in oxidatively stressed primary hippocampal neurons and different strains of senescence-accelerated mice, suggesting its potential role in the pathogenesis of NDs (Zhang et al., 2014). Xie et al. (2015) assayed levels of miR-130b and other AD-related miRNAs in serum of patients with mild cognitive impairment (MCI); based on their results, miR-130b was not deregulated in MCI patients with respect to normal controls.

### Circulating miRNAs in VaD, Their Downstream mRNA Targets, and Corresponding Gene Network

**Figure 5:**
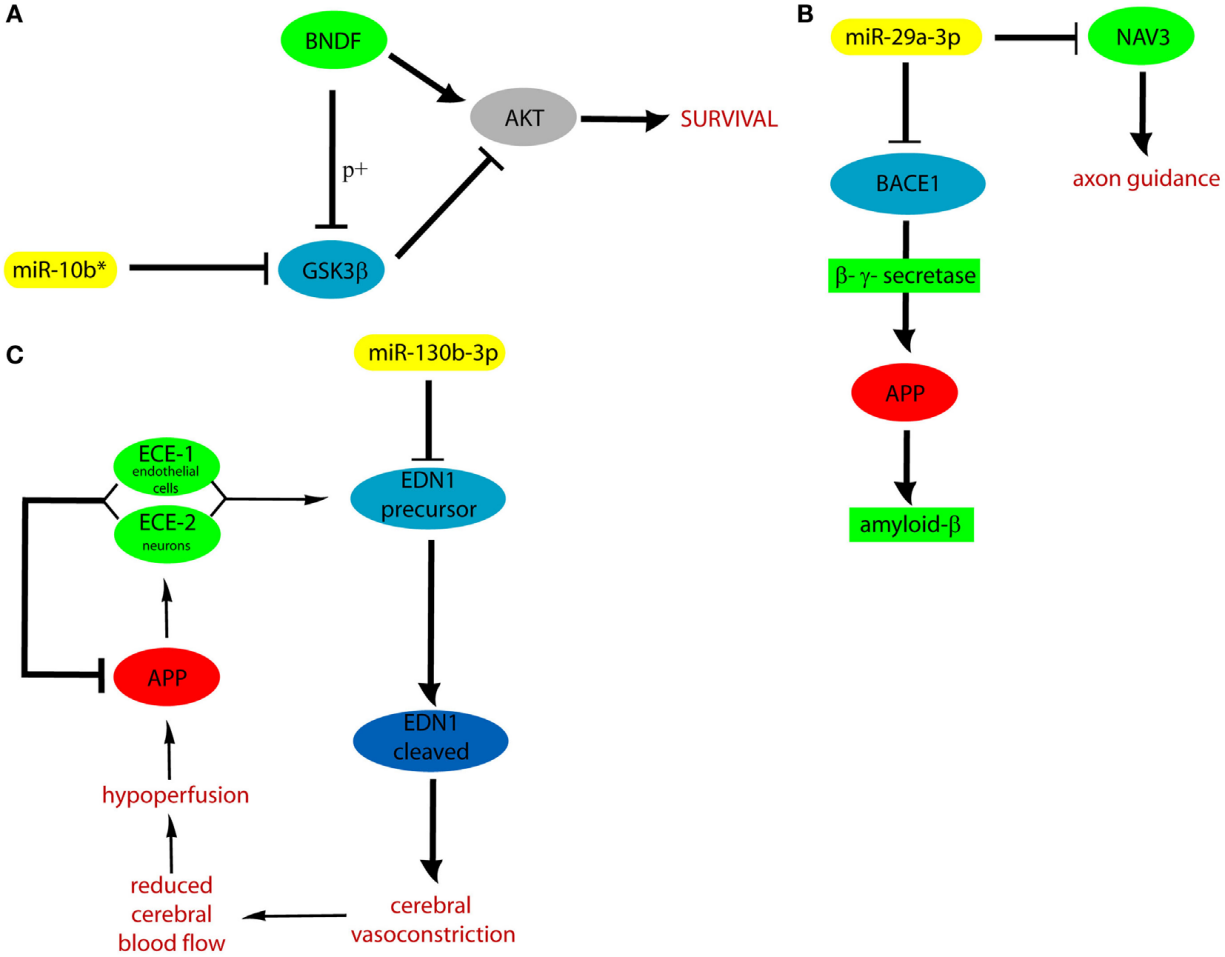
Cellular pathways controlled by miR-10b* (A), miR-29a (B), and 130b-3p (C). APP, amyloid precursor protein; ECE, endothelin converting enzyme; p+, phosphorylation.

We hypothesized that DE cmiRNAs detected in our experiments could shed a light on the genes and pathways involved in VaD, which remain still elusive. Indeed, our computational analysis on validated and predicted miRNA targets allowed us to discover that miR-10b*, miR-29 family, and miR-130b-3p perform important functions in regulating CNS activity and also are involved in neurodegenerative and cardiovascular diseases: it is worth to stress that both CNS and cardiovascular system are involved in VaD. Among miR-10b* targets, Glycogen synthase kinase3-β (GSK3β) is involved in mechanisms underlying learning and memory. It also is involved in local responses to cerebral inflammatory processes (Llorens-Martín et al., 2014). Moreover, GSK3β overexpression inhibits brain-derived neurotrophic factor (BDNF)-mediated survival pathway (Liu et al., 2015). GSK3β is comprised in six and four of the pathways listed by the DIANA mirPath and GeneTrail, respectively (Figure 3). The miR-29 family comprises three members (i.e., miR-29a, miR-29b, and miR-29c) that have important roles in both nervous and cardiovascular systems (Kriegel et al., 2012). A significant negative correlation was detected between miR-29a-3p and Aβ42 peptide in CSF and blood from AD patients (Hébert et al., 2008). Expression of miR-29a/b-1 cluster was found to be significantly decreased in AD patients, coupled to abnormally high levels of BACE1 protein (Hébert et al., 2008). Similar correlations between expression of this miRNA cluster and BACE1 were found during brain development and in primary neuronal cultures (Hébert et al., 2008). Notably, our data showed significant negative correlation between miR-29a expression and cognitive impairment in VaD patients. Neuron navigator 3 (NAV3), involved in axon guidance, is a very important target of miR-29a-3p: underexpression of miR-29a-3p affects neurodegenerative processes by enhancing neuronal NAV3 expression in AD brains (Shioya et al., 2010). The potentially important pathogenetic role of miR-130b-3p in VaD is suggested by the biomolecular functions of its targets. EDN1 is the most powerful among the three members of the endothelin family (EDN1, EDN2, and EDN3) (Maguire and Davenport, 2014). They are synthesized mainly in the endothelium and perform a key role in the homeostasis of the vascular system, acting as powerful vasoconstrictor agents (Agapitov and Haynes, 2002). Endothelins are primarily involved in cerebral circulation deficits and in the pathogenesis of many heart and circulatory system diseases (Agapitov and Haynes, 2002). Another relevant target of miR-130b-3p is ENPP5: it is highly expressed in the brain and plays an important role in communication among neuronal cells (Ohe et al., 2003). Its overexpression is important after the onset of neuronal damage, when CNS attempts to reestablish lost neuronal connections (Schinelli, 2006). Both EDN1 and ENPP5 functions would agree with potential involvement of miR-130b-3p underexpression in contributing to neurodegeneration. It is worth to stress that miR-130b-3p, downregulated in both VaD and AD with respect to NCs, was able to discriminate the two diseases with 70% of sensitivity and 46% of specificity, and its expression was negatively correlated with intellectual deterioration in AD. In Figure 5, the potential cellular pathways controlled by miR-10b*, miR-29a-3p, and 130b-3p are depicted, based on their interactions with targets reported above.

## Conclusion

Our study describes a set of non-invasive miRNA biomarkers, which are detectable in plasma of VaD patients, and could confer molecular precision and improve detection power of VaD diagnosis. The use of cmiRNAs as biomarkers should be effectively associated with other traditional well validated markers of VaD (e.g., structural and molecular imaging). Further multicentric studies on larger cohorts of patients and on other NDs will be needed to verify the effective diagnostic and discriminatory power of miR-10b*, miR-29a-3p, and 130b-3p and other cmiRNAs in VaD and AD. Profiling of circulating ncRNAs in NDs could have an important impact on clinical practice in Neuropsychiatry and also be exploited to improve our understanding of still partially characterized molecular aspects of these phenotypes (Geekiyanage et al., 2012; Geaghan and Cairns, 2014).

## Author Contributions

MP conceived and coordinated the project with the critical collaboration of MR. MP, MR, PB, ME, MS, and CP designed experiments; LT, MT, and DB performed them. RS carried out patient’s recruitment and clinical data analysis. CB and AC performed computational analysis. LT realized statistical analysis. MP, MR, and LT wrote the paper. All authors contributed to the critical revision of the data, read, and approved the final manuscript.

## Conflict of Interest Statement

The authors declare that the research was conducted in the absence of any commercial or financial relationships that could be construed as a potential conflict of interest.

## Footnotes

^1^www.tm4.org

^2^http://mirtarbase.mbc.nctu.edu.tw

^3^http://starbase.sysu.edu.cn/

^4^http://diana.imis.athena-innovation.gr/DianaTools/index.php?r=microT_CDS

^5^http://genetrail.bioinf.uni-sb.de

^6^http://diana.imis.athena-innovation.gr/DianaTools/index.php?r=mirpath/index

^7^http://diana.imis.athena-innovation.gr/DianaTools/index.php?r=tarbase/index

^8^http://chianti.ucsd.edu/cyto_web/plugins/displayplugininfo.php?…MiMIplugin

^9^www.cytoscape.org

^10^http://apps.cytoscape.org/apps/centiscape
