# Supplementary material for: miRNAs Plasma Profiles in Vascular Dementia: Biomolecular Data and Biomedical Implications
Source: Front Cell Neurosci. 2016 Mar 1;10:51. doi: 10.3389/fncel.2016.00051 (PMC4771726; doi:10.3389/fncel.2016.00051)
Supplement: Supplementary file 1 [file Table_1.DOC]

**Table S1. Clinical phenotypes of VaD patients**

| **Type of VaD** | **Number of patients** | **MMSE** | |
| --- | --- | --- | --- |
|  |  | Mean | standard deviation |
| Cortical Multi-Infarct | 16 | 15 | 6.03 |
| Strategic Infarct | 3 | 18 | 0 |
| Subcortical | 19 | 12.86 | 5.54 |

**Table S2. Clinical parameters of patients and controls**

| **Patients and Controls** | **Total**  **Cholesterol** | | **Hypertension** | | **Diabetes** | | **Cigarette Smoker** | | **Carotid Atheromatous Disease** | | **Atrial Fibrillation** | |
| --- | --- | --- | --- | --- | --- | --- | --- | --- | --- | --- | --- | --- |
|  | Mean | Standard Deviation | **(%)** |  | **(%)** |  | **(%)** |  | **(%)** |  | **(%)** |  |
| **VaD** | 182.237 | 51.6482 | 73.68 |  | 39.47 |  | 34.21 |  | 60.53 |  | 10.53 |  |
| **AD** | 192.875 | 45.2119 | 75 |  | 20 |  | 17.5 |  | 25 |  | 5 |  |
| **NCs** | 207.1 | 38.735 | 70 |  | 15 |  | 30 |  | 7.5 |  | 3 |  |

**Table S3. Primer sequences**

| **Target** | **Forward** | **Reverse** | **amp. length** |
| --- | --- | --- | --- |
| BACE1 | GATCATTGTGCGGGTGGAGA | CTTTCTTGGGCAAACGAAGGT | 116 |
| CCT5 | TCAGACAGCAAAAACCACGC | TCAACGTCTCTCCGCTCCAT | 117 |
| EDN1 | TCAGCAGTTAGTGAGAGGAAGA | TTGCCTTTCAGCTTGGGATCAT | 123 |
| GSK3B | CGAGAGCTCCAGATCATGAGAA | CGGAACATAGTCCAGCACCA | 123 |
| ITPR1 | CGGAGCAGGGTATTGGAACA | CTGAGGGCTGAAACTCCAGG | 116 |
| LPL | TTGGAGAAGCTATCCGCGTG | CGTGGGAGCACTTCACTAGC | 72 |
| NAV3 | TACCAGACACAGCAACTTCCC | CCACTGCTCACTGAACTGCT | 100 |

**Table S4**. Targets of differentially expressed miRNAs in VaD and AD.

| **miR-10b-3p** | **Target Type** | **miR-29a-3p** | **Target Type** | **miR-130b-3p** | **Target Type** |
| --- | --- | --- | --- | --- | --- |
| MARCH5 | P | ABL1 | V | CSF1 | V |
| ATP11B | P | ADAMTS9 | V | DICER1 | V |
| AZI2 | P | **BACE1** | V | PPARG | V |
| BACH1 | P | BCL2 | V | RUNX3 | V |
| C14orf101 | P | BCL7A | V | TP53INP1 | V |
| CCDC68 | P | CD276 | V | UVRAG | V |
| **CCT5** | P | CDC42 | V | **ZEB1** | V |
| CNTLN | P | CDK6 | V | **ACSL4** | P |
| DBF4 | P | **COL4A1** | V | BLCAP | P |
| DOPEY2 | P | COL4A2 | V | BTG1 | P |
| EDARADD | P | CPEB3 | V | **EDN1** | P |
| **FGF13** | P | CPEB4 | V | **ENPP5** | P |
| FMR1 | P | DICER1 | V | HBP1 | P |
| **GLS** | P | DKK1 | V | **ITPR1** | P |
| **GSK3B** | P | DNMT3A | V | MAP7 | P |
| HLA-B | P | DNMT3B | V | MBNL1 | P |
| HOXD1 | P | FGA | V | MLL3 | P |
| **KCNJ3** | P | FGB | V | **SLC44A1** | P |
| KPNA3 | P | FGG | V | TSC22D1 | P |
| LMO3 | P | GLUL | V | ZFYVE26 | P |
| MAX | P | HBP1 | V | ZNF800 | P |
| MBNL2 | P | IMPDH1 | V |  |  |
| MCTP1 | P | ITGA11 | V |  |  |
| MEF2C | P | ITIH5 | V |  |  |
| MLLT10 | P | KREMEN2 | V |  |  |
| MSI2 | P | **LPL** | V |  |  |
| NEK9 | P | MCL1 | V |  |  |
| NEUROD1 | P | MYCN | V |  |  |
| PHKB | P | NASP | V |  |  |
| PNN | P | **NAV3** | V |  |  |
| PPP1CC | P | PIK3R1 | V |  |  |
| PPP3CC | P | PPM1D | V |  |  |
| RAP1A | P | PPP1R13B | V |  |  |
| ROCK2 | P | PTEN | V |  |  |
| RSBN1 | P | PXDN | V |  |  |
| RSPO2 | P | RAN | V |  |  |
| SLC48A1 | P | **S100B** | V |  |  |
| TBC1D9 | P | SAPCD2 | V |  |  |
| TFAP2C | P | SERPINB9 | V |  |  |
| TOR1AIP1 | P | SFRP2 | V |  |  |
| TSGA10 | P | SPARC | V |  |  |
| TTC33 | P | TET1 | V |  |  |
| UBR3 | P | TNFAIP3 | V |  |  |
| XIRP2 | P | AMMECR1L | P |  |  |
| ZNF148 | P | E2F7 | P |  |  |
| ZNF474 | P | ELF2 | P |  |  |
| PAX6 | P | FSTL1 | P |  |  |
| GOLGB1 | P | IREB2 | P |  |  |
| HHIP | P | KDM5B | P |  |  |
| NAV1 | P | PDHX | P |  |  |
| NCOA1 | P | **SIKE1** | P |  |  |
| PCGF5 | P | TMTC3 | P |  |  |
| SPA17 | P |  |  |  |  |
| SPATA5 | P |  |  |  |  |
| WASL | P |  |  |  |  |

Validated (V) and predicted (P) targets of DE miRNAs in VaD and AD. Bold typed targets are involved in relevant biological functions in CNS neurodegenerative diseases.

**Table S5.** Central target nodes of network

| **Gene name** | **Betweenness** | **Closeness** | **Node degree** | **Stress** | **microRNA** |
| --- | --- | --- | --- | --- | --- |
| **ABL1** | 53128.18407 | 0.000194024 | 114 | 1042402 | miR-29a-3p |
| **BCL2** | 43725.92144 | 0.00019685 | 87 | 618550 | miR-29a-3p |
| **CDC42** | 57209.37086 | 0.000184706 | 196 | 1484632 | miR-29a-3p |
| **CDK6** | 3925.902513 | 0.000190223 | 29 | 77466 | miR-29a-3p |
| **DBF4** | 6859.612505 | 0.000187723 | 50 | 122608 | miR-10b-3p |
| **GSK3B** | 65904.27245 | 0.000202881 | 92 | 905754 | miR-10b-3p |
| **HLA-B** | 21479.08582 | 0.000173943 | 33 | 223294 | miR-10b-3p |
| **MAX** | 667705.2549 | 0.000261028 | 825 | 11331412 | miR-10b-3p |
| **MCL1** | 10741.79516 | 0.000188573 | 27 | 112630 | miR-29a-3p |
| **NCOA1** | 12103.90375 | 0.000183857 | 71 | 209502 | miR-10b-3p |
| **PIK3R1** | 61403.80834 | 0.000189934 | 140 | 1242124 | miR-29a-3p |
| **RAN** | 37519.18522 | 0.000175716 | 81 | 781382 | miR-29a-3p |
| **TSC22D1** | 15665.48624 | 0.000181984 | 28 | 143384 | miR-130b-3p |
| **WASL** | 22223.47917 | 0.000202963 | 34 | 273272 | miR-10b-3p |
